# Unravelling prostate cancer risk and protective factors among urology patients in a Tanzanian population

**DOI:** 10.3389/fonc.2026.1696848

**Published:** 2026-03-19

**Authors:** Fidelis Charles Bugoye, Richard Biegon, Nazima Dharsee, Fidelice Mafumiko, Herry Kibona, Patrick I. Chiyo, Kirtika Patel, Simeon Mining, Rispah Torrorey-Sawe

**Affiliations:** 1 Directorate of Forensic Science and DNA Services, Government Chemist Laboratory Authority, Dar es Salaam, Tanzania; 2 Department of Pathology, Moi University, Moi Teaching & Referral Hospital, Eldoret, Kenya; 3 Clinical Research, Training and Consultancy Unit, Ocean Road Cancer Institute, Dar es Salaam, Tanzania; 4 Department of Clinical Oncology, Muhimbili University of Health and Allied Sciences, Dar es Salaam, Tanzania; 5 Department of Urology, Muhimbili National Hospital, Dar es Salaam, Tanzania; 6 Wildlife Genetics and Forensics Laboratory, Kenya Wildlife Service, Nairobi, Kenya; 7 Department of Biology, Duke University, Durham, NC, United States

**Keywords:** MNH, modified and non-modified risk factors, Ocean Road Cancer Institute, ORCI, prostate cancer, Sub-Saharan African, Tanzanian population

## Abstract

**Background:**

Prostate cancer (PCa) is a leading cause of cancer-related morbidity and mortality among men globally. The prevalence is disproportionate among men of African descent and more specifically, in East Africa, where it is characterized by aggressive tumour biology and poor survival outcomes. Despite its high burden, the risk factors underlying its disproportionate prevalence remain understudied in this population. This study investigated lifestyle risk and protective factors among prostate cancer (PCa) patients, including demographic, dietary, lifestyle, and family cancer history, at Muhimbili National Hospital (MNH) and Ocean Road Cancer Institute (ORCI) in Dar es Salaam, Tanzania.

**Methods:**

This case-control study compared PCa patients with non-PCa controls. Data on sociodemographic, lifestyle, diet, and family history were collected using a standardized questionnaire. Multivariate logistic regression identified significant risk and protective factors for PCa from each of these factors. Several statistical approaches were used to rank a Tanzanian urban population’s significant risk or protective factors.

**Results:**

Ranking of broad classes of factors revealed that diet, lifestyle, sociodemographic, family, and patient history of cancer and other disease factor groups, in order of importance, were associated with PCa in men. However, the single best model explaining the odds of being PCa had intake of red meat, coffee, alcohol, tomato, and marital status as independent variables. Specifically, increased intake of red meat (AOR = 5.248), and alcohol (AOR = 2.189) were associated with a high PCa incidence while increased intake of soya (AOR = 0.248), coffee (AOR = 0.603), tomato (AOR = 0.188), and not being married (AOR = 0.147) were associated with lower incidence of PCa in the Tanzanian urban population.

**Conclusion:**

The findings suggest that dietary and lifestyle factors have a significant association with PCa incidence in a Tanzanian population compared to sociodemographic, family cancer history, and exposure to infectious and other lifestyle diseases. We recommend further research, including prospective studies or randomized controlled trials with large sample sizes, to confirm these findings, as they suggest health initiatives for the prevention of PCa among high-risk populations, such as urban male populations.

## 1 Introduction

Prostate cancer (PCa), the most common cancer in men globally, presents a severe public health burden, especially in ageing populations. While PCa poses a growing global health burden, sub-Saharan Africa (SSA) bears a disproportionate share of its impact, with men of African descent experiencing some of the highest mortality rates worldwide (1, 2). In Tanzania, PCa is the most common cancer among men, with a prevalence of 19.3 per 100,000 population, an incidence rate of 8.8%, and a mortality rate of 7.4% (3, 4). The prevention of PCa is still challenging to achieve despite research advancements, and the incidence and fatality rates of PCa vary significantly between populations (5). However, if important risk factors are identified and adequately managed, a considerable reduction in disease incidence and mortality can be realised (6). Understanding these risk and protective factors can help guide clinical care, public health programs, and awareness-raising efforts to reduce PCa mortalities (6, 7). Despite advancements in PCa diagnosis and treatment, the role of both modifiable and non-modifiable risk factors, including socioeconomic status, dietary habits, lifestyle choices, and genetic predisposition, is fragmented and incomplete in an African population. There is limited knowledge on how socioeconomic position, food patterns, lifestyle decisions, family history of cancer, and medical condition interact in Sub-Saharan Africa and other low- and middle-income countries (5, 8). Furthermore, modifiable risk factors may exhibit ethnic, racial, or regional disparities influenced by variations in dietary patterns and lifestyle behaviours. Such differences highlight the potential for tailored interventions targeting specific demographic or geographic populations to enhance public health outcomes (9, 10). Because of the varied genetic origins of the populations under study, researchers have reported conflicting results about the relationship between risk factors and PCa (11, 12). Notably, these studies continue to show a marked under-representation of males from East African populations. To fill in these gaps and offer insights into East African populations, further research is desperately needed, especially within the Tanzanian population. These intricate risk factors were investigated in this study within the Tanzanian population, filling important knowledge gaps and opening the door to more individualised preventative measures and better clinical results.

To the best of our knowledge, this is the first study to incorporate statistical ranking metrics such as the Akaike Information Criterion (AICc) to discern the importance of different factors that may be protective or risks for PCa in an urban male population in Tanzania. In addition, the association between the incidence of PCa and several variables, including dietary and lifestyle choices, demographic characteristics, and a family history of PCa, in the Tanzanian population contributes to knowledge of the epidemiology of PCa in an African population, and it may serve as a guide for tailored therapies and public health policies that aim to lower the burden of disease in low to middle-income countries.

## 2 Subjects and materials

### 2.1 Study design

An unmatched case-control study was conducted from May 2023 to May 2024 at Muhimbili National Hospital (MNH) and Ocean Road Cancer Institute (ORCI). MNH and ORCI are Tanzania’s leading national tertiary referral hospitals, providing specialised care to a broad and ethnically diverse patient population. The study population consisted of male patients visiting the urology clinics in these hospitals.

### 2.2 Sample size calculation

The sample size for this case-control study was determined using the *EpiTools* web-based calculator (13). We assumed an odds ratio (OR) of 2.6, 80% power, and a 95% confidence level, and based on an exposure prevalence of 20% in controls, the estimated total sample size required was 170 participants (85 cases and 85 controls). This effect size was selected based on prior epidemiological studies in Sub-Saharan Africa, where moderate associations between PCa and common risk factors, including lifestyle, dietary habits, and ageing, have been reported (2, 14, 15). The calculation ensures sufficient power to detect clinically relevant associations and the high PCa burden in Sub-Saharan Africa (8, 16).

### 2.3 Inclusion criteria

Participants were selected based on specific criteria to ensure the validity and reliability of the study findings. Cases were comprised of men with histopathologically confirmed PCa, as definitive tissue diagnosis remains the gold standard for oncologic confirmation. To further refine the case group and improve clinical relevance, inclusion was restricted to those with a pre-diagnosis Prostate-Specific Antigen (PSA) level greater than 4 ng/mL, a common clinical threshold prompting biopsy. The control group consisted of participants with a histologically confirmed absence of PCa, typically from biopsy specimens. Importantly, to maintain diagnostic certainty and data integrity, any potential participants with unconfirmed PSA or incomplete histological verification records were excluded from the study. Furthermore, only patients who provided written informed consent were enrolled, reinforcing ethical compliance and minimising selection bias.

### 2.4 Sampling procedure

Participants for this study were selected from MNH and ORCI using purposive sampling. A total of 170 participants (85 PCa cases and 85 controls) were enrolled after providing written informed consent, adhering to the study’s predefined eligibility criteria. This 1:1 case-control ratio was selected to optimise statistical power and ensure balanced group comparisons, thereby enhancing the validity of risk factor analyses.

### 2.5 Data collection tool

A standardised, pre-tested questionnaire was used to systematically collect data on potentially risk or protective factors for PCa. The questionnaire tool was designed to capture five primary exposure domains: sociodemographic characteristics (including age at diagnosis, monthly income), dietary patterns (including frequency of red meat, dairy, vegetable, and soy consumption quantified in standardised servings per week), lifestyle behaviours (encompassing physical activity frequency per week, as well as the frequency of alcohol and coffee intake), family cancer history and relevant medical history, particularly diabetes mellitus, gonorrhoea infection, and other pertinent comorbidities. The standardized questionnaire utilised frequency metrics to ensure quantitative precision in exposure assessment, thereby enabling robust dose-response assessment.

### 2.6 Statistical analyses

A Generalized Linear Model (GLM) with a binomial error structure and a logit link function was used to assess the association between PCa incidence and various potential risk and protective factors. The response variable was binary, with 1 indicating the presence of PCa (for the case group) and 0 indicating the absence of PCa (for the control group). Risk and protective factors were grouped into dietary, lifestyle, sociodemographic, disease exposure, and family cancer history factors and were included as independent variables. Most of our independent variables (dietary factors, income, frequency of smoking, and exercise were rank-ordered into a five-point ordinal scale with “distance” between categories assumed consistent and a single coefficient estimated to represent the average change in the log-odds for every one-unit increase in the rank. The rest of the independent variables (gender, marital status, education), except occupation, were converted into “dummy” binary variables to reduce sparseness in some categories within variables.

First, bivariate GLM analyses were conducted for each variable within the respective groups. Subsequently, multivariate GLM analyses were performed for each group, evaluating all possible covariate combinations using the MuMIn R package (17). The best model of all possible combinations of variables for each group was selected based on the Akaike Information Criterion (AIC). Akaike Information Criterion (AIC) was used to select the best-performing model from a set of candidates by balancing model fit with simplicity, effectively preventing overfitting. It estimates the relative information lost by a model, with lower AIC values indicating better quality, making it ideal for comparing models across various statistical applications. The AIC-selected best models were the most parsimonious with greater explanatory power following a penalty for model overfitting. Model performance was assessed using the area under the curve (AUC) of the receiver operating characteristic (ROC) curve, which measures predictive accuracy. AUC values range from 0.5 (no predictive power) to 1.0 (perfect prediction), with values above 0.80 considered clinically useful (18, 19).

Additionally, pseudo-R² measures (McFadden’s, Maximum likelihood, Nagelkerke’s, and Coefficient of Discrimination) were calculated to evaluate model fit (20). For instance, McFadden’s R² values between 0.2 and 0.4 indicate a strong fit. Model fit and selection metrics, including AIC, AUC, and pseudo-R², were used to rank the importance of risk factor groups in predicting PCa in a Tanzanian urban male population. All analyses were conducted using R software (21). We also calculated the Odds ratio (OR) for each variable in the model. The OR = 1 was interpreted as no association, the OR > 1 was interpreted as a positive association, and the OR < 1 was interpreted as a negative association. Generally, larger absolute values (either greater than 1 or less than 1) suggest a stronger association. To handle unstable estimates caused by “separation,” which occurs when predictor variables perfectly predict the binary outcome, causing maximum likelihood estimates for coefficients to approach infinity and algorithms to fail to converge, we conducted a penalized likelihood using Firth’s bias reduction method (22). All factors (dietary, lifestyle, sociodemographic, disease exposure, and family cancer history) tested were combined in a single multivariate GLM analysis, and to minimize the issues of multicollinearity and separation, we used Firth penalized likelihood and model selection using forward and backward model selection in the R software for statistical computing.

## 3 Results

### 3.1 Socio-demographic factors

Prostate cancer (PCa) Case and Control patient groups had an average age of 71.77 ± 8.53 and 68.87 ± 7.20 years, respectively (**Table 1**). Physical health characteristics among all recruited participants reveal a mean ± SD BMI of 23.87 ± 3.33 and 23.12 ± 7.51 for case and control group participants, respectively (**Table 1**). Most of the PCa case group had a mean ± SD of 5.32 ± 2.72 number of children, compared with 4.27 ± 2.47 for the control group. The majority of men (54.44%, N = 169) sampled had an average monthly income of Tanzanian shillings (T.Sh). 300,000 (~USD 115) and below (**Table 2**).

**Table 1:** Biometric and demographic characteristics of PCa cases and controls in a Tanzanian urban population.

Variable	Unit of measurement	PCa controls mean ± SD	PCa cases mean ± SD
Age	Years	68.87 ± 7.20	71.77 ± 8.53
Height	Meters (M)	1.68 ± 0.07	1.66 ± 0.07
BMI	–	23.87 ± 3.33	23.12 ± 7.51
Weight	Kilograms (Kg)	68.0 ± 6.64	69.19 ± 9.60
Number of Children	–	4.27 ± 2.47	5.32 ± 2.72

**Table 2:** PCa incidence across several sociodemographic groups among urban males in Tanzania.

Variable	Number of PCa controls, n (%)	Number of PCa cases n (%)	Total number, N (%)
Marital status			
Married	50 (39.68)	76 (60.32)	126 (74.56)
Other	32 (74.42)	11 (25.58)	43 (25.44)
Education level			
Higher	21 (47.73)	23 (52.27)	44 (26.04)
Lower	61 (48.80)	64 (51.20)	125 (73.96)
Children (present or absent)			
Children absent	7.0 (63.64)	4.0 (36.36)	11 (6.51)
Children present	75 (47.47)	83 (52.53)	158 (93.49)
Occupation			
Agriculture	13 (48.15)	14 (51.85)	27 (15.98)
Business entrepreneur	16 (44.44)	20 (55.56)	36 (21.30)
Employed	13 (61.90)	8.0 (38.10)	21 (12.43)
Peasant	15 (51.72)	14 (48.28)	29 (17.16)
Retiree	25 (44.64)	31 (55.36)	56 (33.14)
Monthly income in 1000s of shillings			
Up to 300	38 (41.30)	54 (58.70)	92 (54.44)
301 - 800	24 (46.15)	28 (53.85)	52 (30.77)
801 - 1,500	13 (76.47)	4 (23.53)	17 (10.06)
1,500 - 2,000	5.0 (100.0)	0.00 (0.00)	5.0 (2.96)
2,001 and greater	2.0 (66.67)	1.0 (33.33)	3.0 (1.78)

The prostate cancer case group had the most married men (60.32%), whereas the control group had only 25.58% of the respondents who were married, indicating that the incidence of PCa was higher among married men than men in the other-marital category, including single, divorced, and widowed (**Table 2**). On the other hand, the majority (74.42%) of men in the other-marital status category were from the case-control group, and only 39.68% were from the PCa case group. Most patients (85.21%) had a monthly income of Tsh. 800,000 (~USD 300) or less, and the rest had an income of more than Tsh.800,000 (~USD 300). The percentage of patients in the control group with a monthly income of less than 800,000 was 75.61 and 24.39 with an income of more than 800,000. On the other hand, most patients (94.12%) in the case group had a monthly income of less than Tsh. 800,000, whereas only 5.88% had a monthly income greater than Tsh. 800,000.

In terms of education, the majority (73.96%) of the respondents, including both cases and control recruited participants were characterized by lower education levels (no education, primary education, and secondary education) combined, but the proportion of professionals with higher education levels (Diploma and university degree holders) was slightly above one-quarter (26.04%) of the study population. The percentage of patients in each education category was similar among the cases and control patient groups (**Table 2**). For example, 52.27% and 47.73% of the patients with higher education levels were from cases and control groups, respectively (**Table 2**).

Sociodemographic variables, including age, marital status, income, and the number of children, were statistically significant predictors of PCa incidence in bivariate GLM models. However, variables such as BMI, educational status, occupation, and the presence or absence of children were not statistically significant in bivariate model analyses (**Supplementary Table 1**).

**Figure 1:**
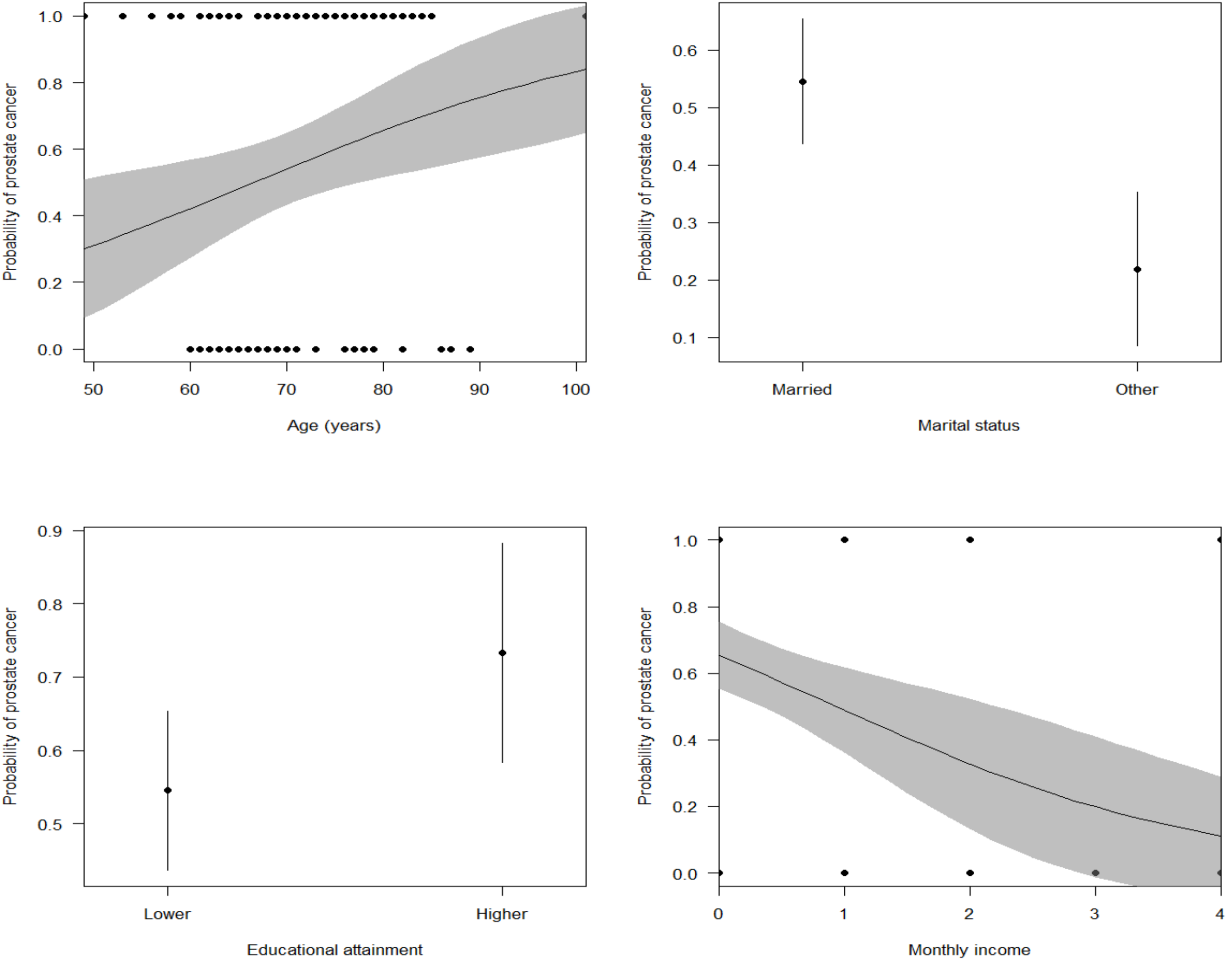
The marginal effects of socio-demographic variables such as age, marital status, educational attainment, and income on the incidence of cancer among urology patients.

In a multivariate analysis, the best sociodemographic and economic model for predicting the incidence of PCa included variables such as marital status, income, age, and the level of education (AUC = 0.745, McFadden’s R^2^ = 0.134, Maximum likelihood pseudo R^2^ = 0.169, Nagelkerke’s R^2^ = 0.226, Coefficient of Discrimination D = 0.173, LRT: χ^2^ = 31.34, P < 0.0001). Specifically, the incidence of PCa significantly reduced with increasing monthly income (**Table 3**, **Figure 1**). In addition, the incidence of PCa increased with an increase in age (**Table 3**, **Figure 1**). The incidence of PCa was higher among individuals who had attained a higher education level (diploma and above) compared to those with a lower education level (secondary education and below) (**Table 2**). Married men also had a higher incidence of PCa than men in other marital categories (divorced, single, and widowed) (**Figure 1**).

**Table 3:** Multivariate logistic regression of socioeconomic and demographic factors associated with the incidence of PCa.

Variable	Estimate	Std. Error	Z Statistic	P- value	AOR estimate	AOR 95% CI
Intercept	-1.923	1.609	-1.2	0.2321		
Age in years	0.048	0.022	2.19	0.0288	1.05	1.01-1.09
Other marital status *cf.* Married	-1.453	0.418	-3.47	0.0005	0.23	0.10-0.52
Lower education *cf.* Higher education	-0.829	0.45	-1.84	0.0658	0.44	0.19-1.09
Monthly income	-0.676	0.244	-2.77	0.0057	0.51	0.32-0.84

### 3.2 Lifestyle factors

There was considerable variation in alcohol consumption among the PCa case and control groups. Specifically, 88.89% of cases reported drinking alcohol 3–4 times per week before their PCa diagnosis, while the majority of the control group (65.69%) reported never consuming alcohol. Similarly, smoking behaviour differed markedly between the two groups. Sixty per cent (60%) of the cases were characterised by heavy smoking, whereas 84.31% of the control participants were nonsmokers. Physical activity levels also showed a notable contrast. Most PCa cases reported never engaging in physical exercise, while most members of the control group (80.95%) reported exercising 3–4 times per week (**Table 4**).

**Table 4:** Prostate cancer incidence across persons with different lifestyle choices.

Variable	Variable status	PCa control group n (%)	PCa case group n (%)	N (%)
Alcohol consumption (times per week)	Never	67 (65.69)	35 (34.31)	102 (60)
	Rarely	11 (52.38)	10 (47.62)	21 (12.35)
	1-2	3 (15.79)	16 (84.21)	19 (11.18)
	3-4	2 (11.11)	16 (88.89)	18 (10.59)
	5-7	0 (0.00)	10 (100.00)	10 (5.88)
Physical activities (times per week)	Never	8 (15.69)	43 (84.31)	51 (30.00)
	Rarely	21 (58.33)	15 (41.67)	36 (21.18)
	1-2	36 (64.29)	20 (35.71)	56 (32.94)
	3-4	17 (80.95)	4 (19.05)	21 (12.35)
	5-7	1 (16.67)	5 (83.33)	6.0 (3.53)
Tobacco smoking	Highly smoker	69 (60.00)	46 (40.00)	115 (67.65)
	Moderate smoker	7 (31.82)	15 (68.18)	22 (12.94)
	Rarely smoker	3 (15.79)	16 (84.21)	19 (11.18)
	Non-smoker	4 (28.57)	10 (71.43)	14 (8.24)
Bicycle riding (kilometres per week)	Never	28 (35.44)	51 (64.56)	79 (46.47)
	Rarely	15 (62.50)	9 (37.50)	24 (14.12)
	1-50	31 (68.89)	14 (31.11)	45 (26.47)
	51-100	5 (33.33)	10 (66.67)	15 (8.82)
	101-150	4 (66.67)	2 (33.33)	6.0 (3.53)
	Above 150	0 (0.00)	1 (100.00)	1.0 (0.59)
Motor-bike riding (kilometres per week)	Never	71 (48.97)	74 (51.03)	145 (85.29)
	Rarely	2 (28.57)	5 (71.43)	7.0 (4.12)
	1-100	5 (55.56)	4 (44.44)	9.0 (5.29)
	101-200	1 (50.00)	1 (50.00)	2.0 (1.18)
	201-300	2 (40.00)	3 (60.00)	5.0 (2.94)
	Above 300	2 (100.00)	0 (0.00)	2.0 (1.18)
Truck driving	No	71 (46.10)	83 (53.90)	154 (90.59)
	Yes	12 (75.00)	4 (25.00)	16 (9.41)
Sexual activity (times/week)	Never	12 (27.27)	32 (72.73)	44 (25.88)
	Rarely	23 (53.49)	20 (46.51)	43 (25.29)
	1-2	37 (69.81)	16 (30.19)	53 (31.18)
	3-4	11 (39.29)	17 (60.71)	28 (16.47)
	5-7	0 (0.00)	2 (100.00)	2.0 (1.18)

Bivariate GLM models revealed that the frequency of alcohol consumption, tobacco smoking, physical activity, truck driving, and bicycle riding were statistically significant predictors of the incidence of PCa. In contrast, the frequency of sexual activity and motorbike riding had no influence on the incidence of cancer among patients from a bivariate model context (**Supplementary Table 2**).

**Figure 2:**
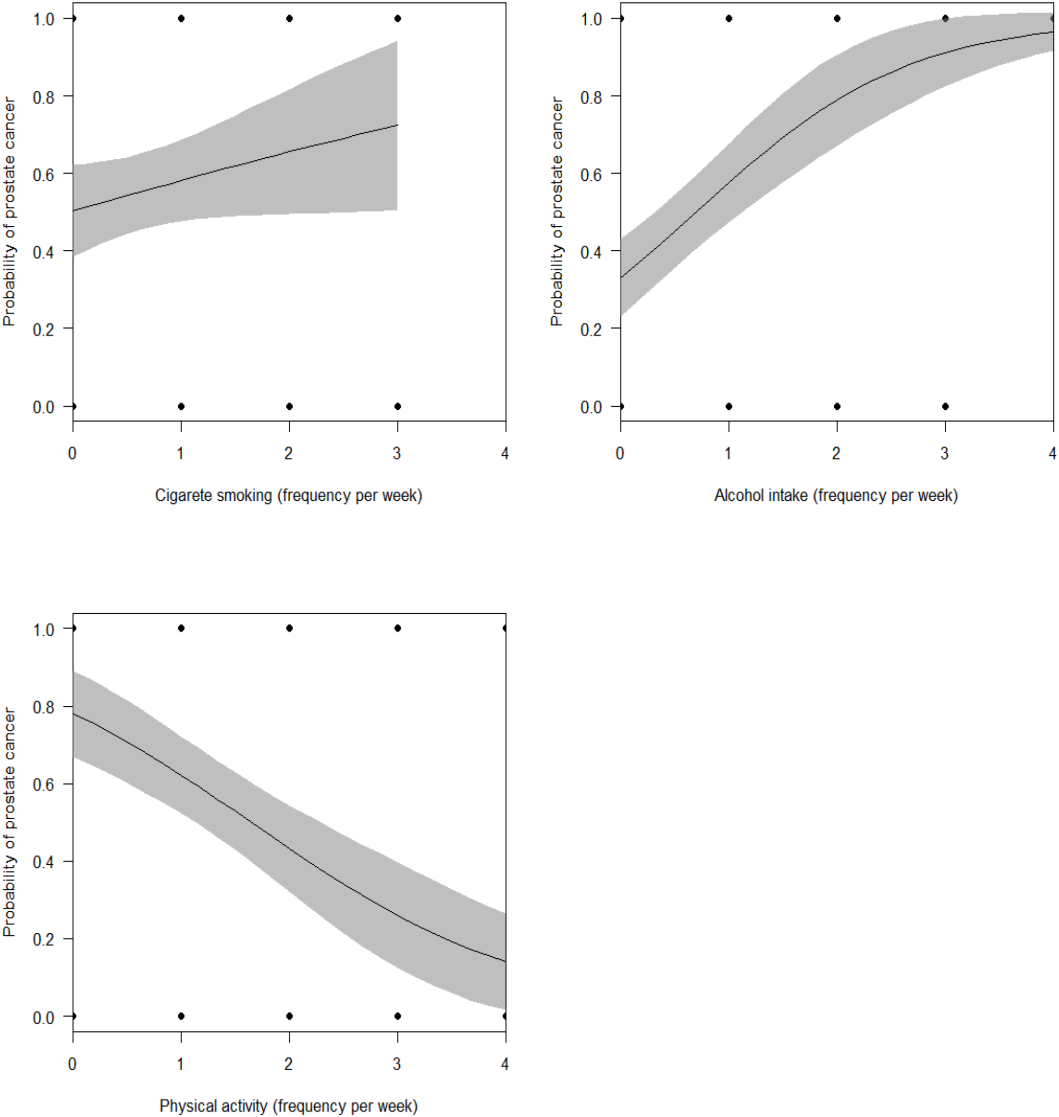
Marginal effects of lifestyle factors such as tobacco smoking, alcohol intake, and physical activity on the incidence of PCa.

In multivariate analyses, the most parsimonious model explaining the incidence of PCa among patients included cigarette smoking, alcohol consumption, and physical activity (AUC = 0.846, McFadden’s R^2^ = 0.292, Maximum likelihood pseudo R^2^ = 0.333, Nagelkerke’s R^2^ = 0.444, Coefficient of Discrimination, D = 0.365, LRT: χ^2^ = 68.74, P < 0.0001). A high frequency of alcohol intake and cigarette smoking were significantly associated with an increased incidence of PCa, while intensity of physical activity was associated with reduced incidence of PCa (**Table 5**, **Figure 2**).

**Table 5:** Multivariate logistic regression of lifestyle factors associated with the incidence of PCa.

Variable	Estimate	Std.Error	z value	P-value	AOR estimate	AOR 95%CI
Intercept	0.17	0.318	0.53	0.590		
Tobacco smoking	0.316	0.217	1.46	0.140	1.37	0.90-2.10
Alcohol	1.012	0.215	4.7	0.000	2.75	1.80- 4.19
Physical activities	-0.772	0.186	-4.15	0.000	0.46	0.32-0.66

### 3.3 Dietary factors

Variation in the reported frequency of consumption of some dietary items was conspicuous between the PCa case group and the PCa control group. The majority of PCa cases (84.85%) reported consuming meat 3–4 times a week before PCa diagnosis, while the majority (90.91%) of the PCa control group never reported red meat consumption. Similarly, the majority (86.36%) of the PCa case group infrequently consumed Soya consumption whereas (65.57%) of the PCa control patients consumed Soya 3–4 times a week. Most of the PCa case patients (85.19%) rarely consumed tomatoes; however, the majority (80.00%) of participants from the control group reported tomato consumption 5–7 times a week. Additionally, most (86.27%) of case participants reported never consuming coffee, and 93.75% frequently consumed foods high in saturated fats before PCa diagnosis. In contrast, 82.50% in the control group reported consuming coffee 3–4 times per week, while 62.30% reported never consuming foods high in saturated fats (**Table 6**).

**Table 6:** PCa incidence across persons with different dietary patterns.

Variable	Variable status	PCa control group n (%)	PCa Case group n (%)	N (%)
Red meat	Never	30 (90.91)	3.0 (9.09)	33 (19.41)
	Rarely	40 (68.97)	18 (31.03)	58 (34.12)
	1–2 times/week	8.0 (22.22)	28 (77.78)	36 (21.18)
	3–4 times/week	5.0 (15.15)	28 (84.85)	33 (19.41)
	5–7 times/week	0.0 (0.00)	10 (100.00)	10 (5.88)
Soya	Never	1.0 (14.29)	6.0 (85.71)	7.0 (4.12)
	Rarely	3.0 (13.64)	19 (86.36)	22 (12.94)
	1–2 times/week	33 (47.14)	37 (52.86)	70 (41.18)
	3–4 times/week	40 (65.57)	21 (34.43)	61 (35.88)
	5–7 times/week	6.0 (60.00)	4.0 (40.00)	10 (5.88)
Tomato	Never	0.0 (0.00)	0.0 (0.00)	81 (47.65)
	Rarely	12 (14.81)	69 (85.19)	29 (17.06)
	1–2 times/week	20 (68.97)	9.0 (31.03)	45 (26.47)
	3–4 times/week	39 (86.67)	6.0 (13.33)	15 (8.82)
	5–7 times/week	12 (80.00)	3.0 (20.00)	51 (30.00)
Coffee	Never	7.0 (13.73)	44 (86.27)	27 (15.88)
	Rarely	12 (44.44)	15 (55.56)	42 (24.71)
	1–2 times/week	28 (66.67)	14 (33.33)	40 (23.53)
	3–4 times/week	33 (82.50)	7.0 (17.50)	10 (5.88)
	5–7 times/week	3.0 (30.00)	7.0 (70.00)	4.0 (2.35)
Vegetables	Never	1.0 (25.00)	3.0 (75.00)	12 (7.06)
	Rarely	2.0 (16.67)	10 (83.33)	34 (20.00)
	1–2 times/week	18 (52.94)	16 (47.06)	55 (32.35)
	3–4 times/week	28 (50.91)	27 (49.09)	65 (38.24)
	5–7 times/week	34 (52.31)	31 (47.69)	15 (8.82)
Dairy food	Never	8.0 (53.33)	7.0 (46.67)	57 (33.53)
	Rarely	29 (50.88)	28 (49.12)	59 (34.71)
	1–2 times/week	32 (54.24)	27 (45.76)	29 (17.06)
	3–4 times/week	11 (37.93)	18 (62.07)	10 (5.88)
	5–7 times/week	3.0 (30.00)	7.0 (70.00)	4.0 (2.35)
Fish	Never	0.0 (0.00)	4.0 (100.00)	42 (24.71)
	Rarely	18 (42.86)	24 (57.14)	65 (38.24)
	1–2 times/week	36 (55.38)	29 (44.62)	33 (19.41)
	3–4 times/week	13 (39.39)	20 (60.61)	26 (15.29)
	5–7 times/week	16 (61.54)	10 (38.46)	33 (19.41)
Green or black tea	Never	0.0 (0.00)	3.0 (100.0)	3.0 (1.76)
	Rarely	1.0 (11.11)	8.0 (88.89)	9.0 (5.29)
	1–2 times/week	17 (53.12)	15 (46.88)	32 (18.82)
	3–4 times/week	53 (63.86)	30 (36.14)	83 (48.82)
	5–7 times/week	12 (27.91)	31 (72.09)	43 (25.29)
Legumes	Never	1.0 (100.00)	0 (0.00)	1.0 (0.59)
	Rarely	3.0 (27.27)	8.0 (72.73)	11 (6.47)
	1–2 times/week	27 (43.55)	35 (56.45)	62 (36.47)
	3–4 times/week	48 (60.00)	32 (40.00)	80 (47.06)
	5–7 times/week	4.0 (25.00)	12 (75.00)	16 (9.41)
Grains	Never	0.0 (0.00)	2 (100.00)	2.0 (1.18)
	Rarely	6.0 (46.15)	7 (53.85)	13 (7.65)
	1–2 times/week	49 (74.24)	17 (25.76)	66 (38.82)
	3–4 times/week	28 (31.46)	61 (68.54)	89 (52.35)
Food containing fats	Never	38 (62.30)	23 (37.70)	61 (35.88)
	Rarely	33 (58.93)	23 (41.07)	56 (32.94)
	1–2 times/week	11 (29.73)	26 (70.27)	37 (21.76)
	3–4 times/week	1.0 (6.25)	15 (93.75)	16 (9.41)
Vitamin-mineral	Never	80 (50.00)	80 (50.00)	160 (94.12)
	Rarely	3.0 (50.00)	3.0 (50.00)	6.0 (3.53)
	1–2 times/week	0.0 (0.00)	2.0 (100.00)	2.0 (1.18)
	3–4 times/week	0.0 (0.00)	2.0 (100.00)	2.0 (1.18)

The bivariate GLM modelling revealed that a high frequency of red meat consumption, consumption of fatty food, and consumption of grains were associated with a higher incidence of PCa among patients (**Supplementary Table 3**). However, a higher frequency of tomato consumption, coffee, and soya consumption was associated with a reduced incidence of PCa among patients (**Table 7**). The frequency of vitamin-mineral supplementation, fish consumption, dairy food consumption, legume consumption, vegetable consumption, and green or black tea consumption did not influence the incidence of PCa among patients in bivariate models (**Supplementary Table 3**).

**Table 7:** Multivariate models for dietary patterns predicting the incidence of PCa.

Variable	Estimate	Std. error	Z value	P-value	AOR estimate	AOR 95% CI
Intercept	1.979	0.973	2.03	0.0421		
Red meat	2.065	0.397	5.20	<0.0001	7.88	3.95 - 19.150
Coffee	-0.561	0.223	-2.51	0.0121	0.57	0.36 - 0.87
Tomato	-1.750	0.359	-4.87	<0.0001	0.17	0.08 - 0.33
Soya	-1.255	0.360	-3.48	0.0005	0.29	0.14 - 0.59
Vitamin-mineral supplements	1.156	1.455	0.80	0.4300	3.18	0.83 - 174.8

**Figure 3:**
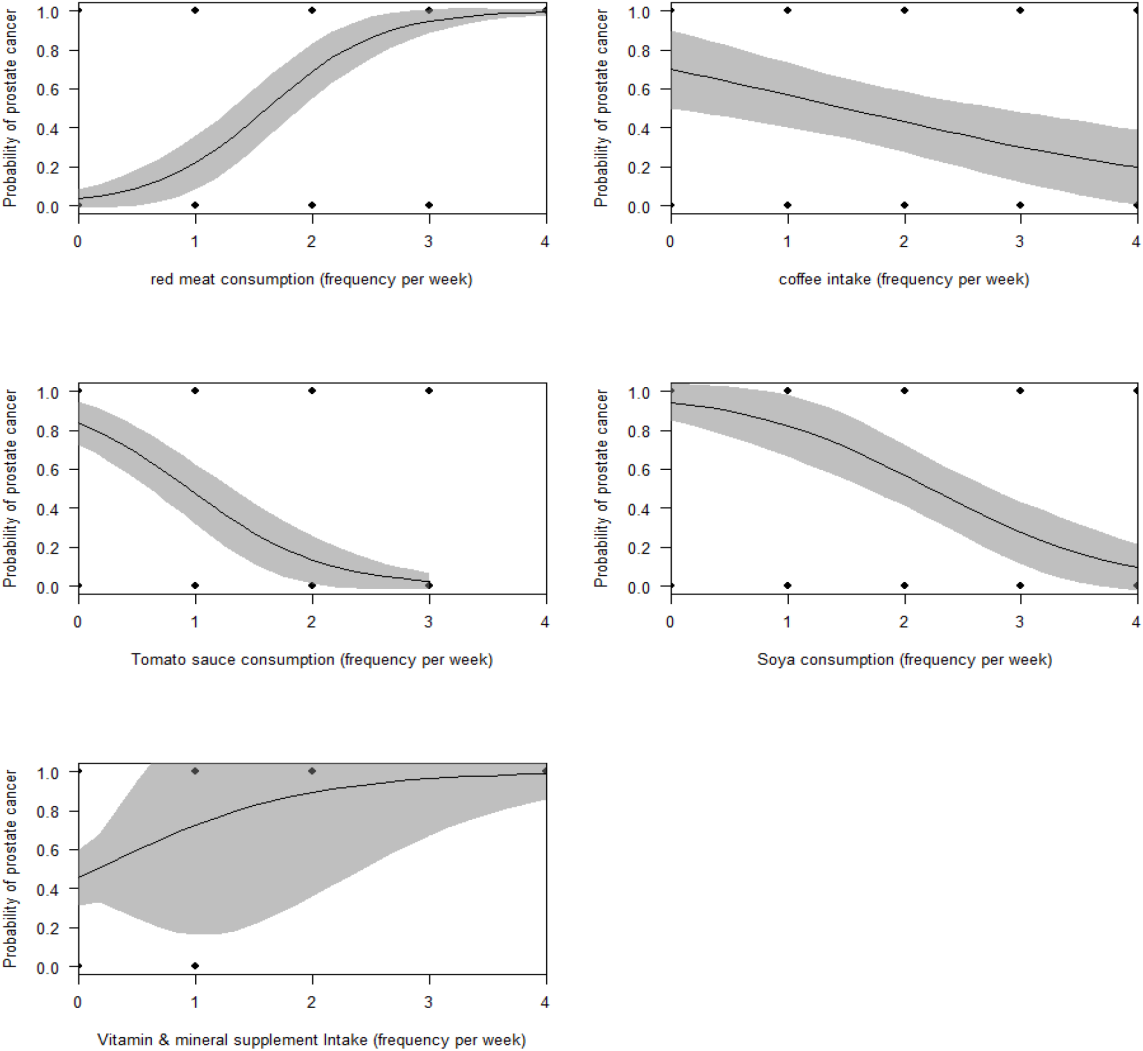
Marginal effects of food and dietary factors on the incidence of PCa.

However, the best model from multivariate analyses revealed that red meat consumption, soya, coffee, and tomato consumption as the best dietary predictors for the incidence of PCa in men (AUC = 0.962, McFadden’s R^2^ = 0.642, Maximum likelihood pseudo R^2^ = 0.589, Nagelkerke’s R^2^ = 0.785, Coefficient of Discrimination, D = 0.696, LRT: χ^2^ = 151.1, P < 0.0001). Specifically, the frequency of red meat consumption was positively associated with PCa incidence. In contrast, the frequency of coffee, soya, and tomato intake was negatively associated with the incidence of PCa (**Table 7**, **Figure 3**).

### 3.4 Exposure to lifestyle and infectious diseases

The frequency of diabetic patients in the PCa control group was 44.85% and 55.15% in the PCa case group, whereas most diabetic patients (64.71%) were in the case-control group and only 35.29% in the PCa cases. Similarly, a higher frequency of patients reporting a history of gonorrhoea (86.67%) was from the PCa case patients, while a few (13.33%) were from the PCa control group before PCa diagnosis (**Table 8**).

**Table 8:** Prostate cancer incidence across participants with different disease statuses.

Variable	Variable status	Control group n (%)	Case group n (%)	N (%)
Diabetes	Negative	61 (44.85)	75 (55.15)	136 (80.00)
	Positive	22 (64.71)	12 (35.29)	34.00 (20.00)
Gonorrhoea	Negative	81 (52.26)	74 (47.74)	155 (91.18)
	Positive	2.0 (13.33)	13 (86.67)	87.0 (51.18)
Syphilis	Negative	81 (50.31)	80 (49.69)	161 (94.71)
	Positive	2.0 (22.22)	7.0 (77.78)	8.00 (4.71)
Other STD	Negative	83 (49.11)	86 (50.89)	169 (99.41)
	Positive	0.0 (0.00)	1.0 (100)	1.00 (0.59)

**Figure 4:**
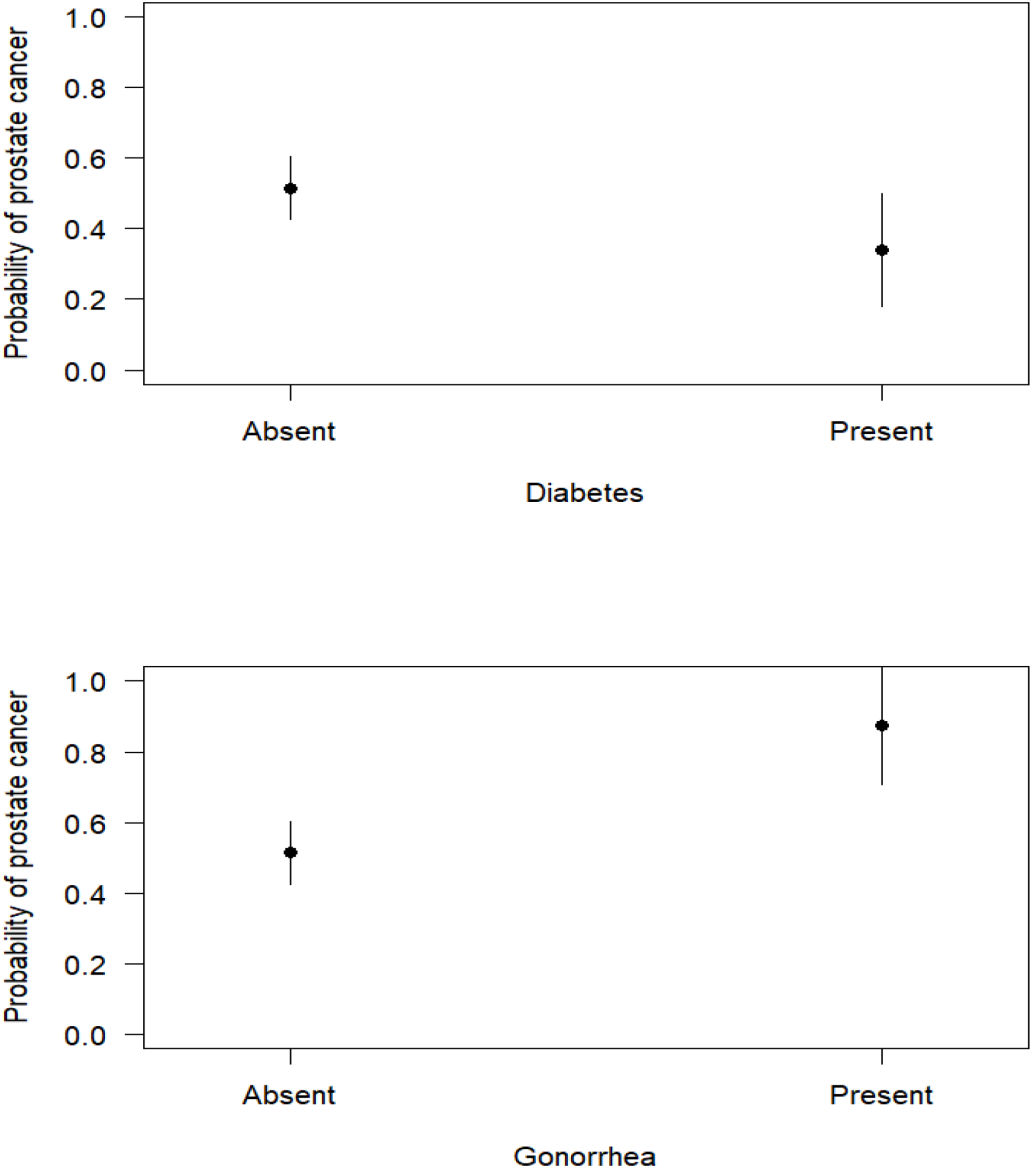
Marginal effects for the influence of disease exposure on PCa incidence.

The bivariate GLM analysis revealed that being diabetic was associated with reduced PCa incidence. In contrast, reported gonorrhoea infection was associated with an increased incidence of PCa. However, syphilis and erectile dysfunction had no influence on PCa among the recruited participants (**Supplementary Table 4**). However, the best multivariate model included diabetes and gonorrhoea as the best disease-explanatory variables for the incidence of PCa in men (AUC = 0.614, McFadden’s R^2^ = 0.053, Maximum likelihood pseudo R^2^ = 0.071, Nagelkerke’s R^2^ = 0.095, Coefficient of Discrimination D = 0.068, Likelihood Ratio Test, LRT: χ^2^ = 12.52, P = 0.00191). The model revealed that exposure to gonorrhoea infections was positively associated with PCa incidence (**Table 9**, **Figure 4**).

**Table 9:** Multivariate models for disease factors predicting the incidence of PCa.

Variable	Estimate	Std. error	Z value	P-value	AOR estimate	AOR 95% CI
Intercept	0.058	0.181	0.32	0.748		
Diabetes	-0.720	0.404	-1.78	0.075	0.487	0.215-1.06
Gonorrhoea	1.874	0.780	2.40	0.016	6.517	1.71- 42.75

### 3.5 Family background with cancer

Regarding the family history of cancer, many patients with a history of PCa (84.62%) were from the PCa case group, and the minority were from the PCa control group, which was at 15.38%. Moreover, patients without a family history of PCa were 55.32% of PCa controls, and 44.68 were from PCa cases (**Table 10**). Among patients with a family history of either breast, ovarian, colorectal, or pancreatic cancer, 100% were PCa cases as compared to 0% of the PCa control group. Additionally, patients with a family history of any other type of cancer apart from either prostate, breast, ovarian, colorectal, or pancreatic cancer were 75.0% of the PCa case group compared to 25% of the PCa case-control group (**Table 10**).

**Table 10:** PCa incidence across participants with different family backgrounds with cancer.

Variable	Variable status	PCa control group n (%)	PCa case group n (%)	N(%)
Family history of PCa	No	78 (55.32)	63 (44.68)	141 (84.43)
	Yes	4.0 (15.38)	22 (84.62)	26.0 (15.57)
Family history of any cancer	No	78 (51.66)	73 (48.34)	151 (90.42)
	Yes	4.0 (25.0)	12 (75.0)	16.0 (9.58)
Family history of breast, ovarian, colorectal, and pancreatic cancer	No	82 (51.57)	77 (48.43)	159 (95.21)
	Yes	0.0 (0.00)	8.0 (100.0)	8.00 (4.79)

Bivariate GLM models for the incidence of PCa among urology patients revealed that a family history of PCa increased the incidence of PCa among patients, while other cancer types, including breast, ovarian, colorectal, and pancreatic cancers, did not influence the incidence of PCa among patients (**Supplementary Table 5**).

**Figure 5:**
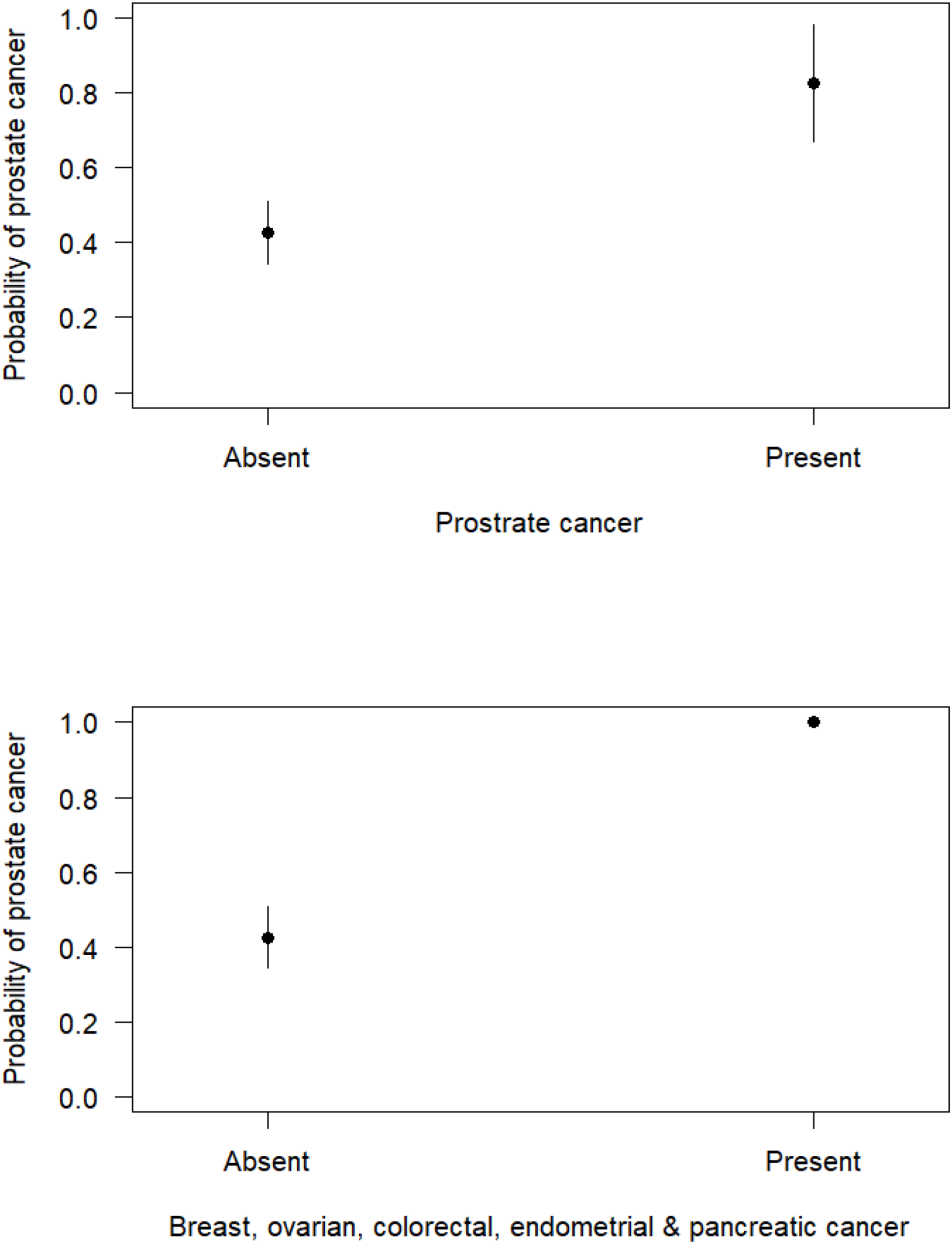
The marginal effects of cancer family history of patients on the incidence of PCa.

However, multivariable GLM models estimated with Firth likelihood and model selection using Akaike Information Criteria (AIC) revealed that the most parsimonious model explaining the incidence of PCa among patients was a model with family history of PCa and family history for either breast, ovarian, colorectal, endometrial, and pancreatic cancers among participants (AUC = 0.637, Coefficient of Discrimination D = 0.116, Wald test = 14.11, P = 0.0009) as covariates (**Table 11**). The inclusion of presence in a family with a history of PCa significantly increased the risk of PCa incidence among patients, as presented in **Table 11** and **Figure 5**.

**Table 11:** The best multivariate model for family history with cancers predicting the incidence of PCa, coefficients estimated using the Firth Penalized likelihood method.

Variables	Estimate	Std. error	χ^2^ value	P-value	AOR estimate	AOR 95% CI
Intercept	-0.294	0.173	2.922	0.0874		
Family history with PCa	1.757	0.547	12.847	0.00034	5.80	2.96- 12.84
Family history with (breast, ovarian, colorectal, endometrial, and pancreatic) cancers	2.7761	1.469	7.037	0.0080	15.81	1.181-2079

### 3.6 The ranking of different factor groups and factors associated with PCa

Based on AIC, the most parsimonious category of models explaining the incidence of PCa is that of the dietary factors, followed by Lifestyle behaviour (**Table 12**). Assuming an AUC of 0.8 has greater power to separate the incidence of PCa, only two risk factor models can be considered, and these include dietary and lifestyle factors. A more complex model, including all potential risk and protective factors and various model selection criteria confirmed the dominance of dietary factors (**Table 13**). Specifically, the model that includes dietary factors such as consumption of red meat, tomato, soya consumption, and coffee intake, and Lifestyle factors like alcohol intake and a sociodemographic factor like marital status (AUC = 0.976, Coefficient of Discrimination, D = 0.755, Nagelkerke R^2^ = 0.1619, McFadden’s Pseudo R^2^ = 0.09418, Wald test = 38.7504, p < 0.0001).

**Table 12:** The ranking of different variables in explaining the incidence of PCa.

Risk factor models	Important variables	AICc	AUC	McFadden’s R^2^	Pseudo R^2^	Nagelkerke’s R^2^	Coefficient of discrimination
Dietary factors	Red meat, Tomato, Coffee, Soya, Vitamin-mineral supplements	96.45	0.962	0.642	0.589	0.785	0.696
Lifestyle behaviours	Alcohol, Smoking, Physical exercise	174.8	0.846	0.292	0.333	0.444	0.365
Social demographic	Age, Marital status, Monthly income, Education level	211.9	0.745	0.134	0.169	0.226	0.173
Family History with Cancer	History with PCa, History with other cancer types	212.8	0.637				0.116
Disease	Diabetes, Gonorrhoea	229.1	0.614	0.053	0.071	0.095	0.068

**Table 13:** The best overall model explaining the incidence of PCa based on selection from all variable categories (diet, lifestyle, sociodemographic, cancer history, and infectious and lifestyle disease exposure) using backward and forward selection and penalized firth likelihood.

Variable	Estimate	Standard error of estimate	Chi-square value	P value	AOR Estimate	AOR 95% CI
(Intercept)	2.895	0.999	9.08	0.0026		
Red meat consumption	1.658	0.357	32.86	0.0000	5.25	2.71 - 12.5
Tomato consumption	-1.672	0.350	31.56	0.0000	0.19	0.08 - 0.36
Soya consumption	-1.396	0.387	15.73	0.0001	0.25	0.1 - 0.52
Alcohol consumption	0.783	0.309	7.28	0.0070	2.19	1.21 - 4.67
Marital status (Other compared to married)	-1.915	0.657	9.02	0.0027	0.15	0.03 - 0.53
Coffee consumption	-0.507	0.231	4.65	0.0311	0.6	0.36 - 0.96

## 4 Discussion

The incidence of PCa is linked to factors, which can be classified into dietary choices, lifestyle behaviour, demographic characteristics, family history of PCa, and exposure to infectious and other lifestyle diseases (23). Multivariate analysis, model ranking using AICc, and AUC, revealed a strong association between dietary and lifestyle factors on the incidence of PCa in an urban Tanzanian population. Other factors found to be moderately associated with the incidence of PCa included sociodemographic factors, family history of cancer diagnosis, and exposure to other infectious and lifestyle diseases. Below, each of the factors associated with PCa in a Tanzanian urban population is discussed in order of importance.

### 4.1 Dietary factors

The dietary model offered an exceptional fit to the data while lowering complexity, as seen by the low AICc found in this study. Furthermore, the strong predictive ability indicated by the high AUC suggests that dietary choices may reliably distinguish between males with and without a high risk of developing PCa. The intake of red meat was positively associated with the incidence of PCa, whilst the intake of tomatoes, soya, vitamin and coffee consumption were associated with reduced PCa incidence in our study population. The positive association between red meat intake and PCa is widely connected to its effect on inflammation and the mechanisms underlying carcinogenic processes during cooking (24). Additionally, nitrates and nitrites in processed meat increase levels of IGF-1, oxidative stress from iron, and hormonal imbalance, which eventually contribute to PCa carcinogenesis (14). Alongside this, our study corroborates evidence that the consumption of red meat increases the risk of PCa (25). For instance, several studies of African populations have reported a strong association between red meat consumption and the increased risk of developing PCa (25). However, the association between red meat and increased PCa risk is still debatable, and inconsistent results have been reported in different populations. The observed disagreement may be linked to various reasons, including variations in study designs, detection bias, and participant selection factors (26).

In contrast to red meat consumption, coffee intake was associated with a low incidence of PCa. Available evidence suggests that the antioxidant and anti-inflammatory properties of coffee, which reduce oxidative stress and inflammation in prostate tissue (27). Similarly, several compounds in coffee affect hormone levels associated with PCa progression, improve insulin sensitivity, assist in regulating glucose levels, and eventually reduce PCa risk (27, 28). In parallel, some coffee compounds can promote apoptosis in cancer cells and prevent carcinogenesis (28). Though inconsistent findings were reported (29), The available evidence from our study agrees with several other studies that demonstrated a negative association between coffee consumption and PCa incidence (27, 30). Likewise, the observed discrepancy may be connected to variations in study designs and participant selection, their different exposure to different environmental factors, and detection bias (26, 31).

Our study also revealed that the frequency of tomato intake was associated with a low incidence of PCa. The results corroborate previous case-control studies, which reported that healthy men who frequently consume tomatoes have a reduced risk of developing PCa compared to men who consume less (14, 32). The anti-carcinogenic effects of lycopene are explained by the antioxidant properties in counterbalancing reactive species and organic free radicals (14). Although our research has demonstrated links between tomato consumption and PCa risk, studies conducted in different populations using different approaches failed to demonstrate the reported inverse relationship (33). The reported inconsistency from other studies confirms the complexity of investigating the influence of dietary lycopene or dietary tomato on PCa risk. Hence, our study suggests that the study investigation of tomato’s influence on PCa should be interpreted with caution, and controlling possible confounders such as lifestyle choices is strongly recommended.

Like tomato intake, soya consumption was associated with low PCa incidence in an urban population in Tanzania. Previous studies have revealed that Daily or frequent consumption (≥1 time/day) is associated with a more pronounced reduction in PCa risk compared to rare consumption, and the protective effect is strongly linked to the consumption of non-fermented soy products in previous studies (34). The primary beneficial components in soy are isoflavones, such as genistein and daidzein, compounds believed to reduce PCa risk through several mechanisms (35, 36). First, they modulate hormone pathways by binding to oestrogen receptors and downregulating androgen receptors and prostate-specific antigen levels, thus mitigating androgen-mediated prostatic stimulation. Second, soy isoflavones can inhibit cancer cell growth by arresting the cell cycle and inducing apoptosis (programmed cell death). Third, isoflavones possess antioxidants and anti-inflammatory effects that protect cells from damage (36).

Last, among dietary factors associated with PCa incidence, the frequency of vitamin-mineral supplementation was associated with a higher incidence of PCa in a Tanzanian urban population. This finding agrees with previous research indicating that frequent use of multivitamins (more than 7 times per week) has been linked to increased susceptibility to prostate cancer (37, 38).

Consumption of fatty food and grains, although associated with a higher incidence of PCa among patients in bivariate analyses, was excluded in a multivariate model of dietary factors associated with PCa incidence. Based on epidemiological studies, high consumption of fat and specific grains may show a positive association with PCa incidence in bivariate analyses, yet these associations frequently disappear or become non-significant in multivariate analyses (39). When adjustments are made for potential confounders such as red meat consumption and total caloric intake, the association between fat/grain consumption and PCa incidence often disappears (39).

In addition, fish consumption, dairy food consumption, other legume consumption, vegetable consumption, and green or black tea consumption did not influence the incidence of PCa among patients in bivariate or multivariate models. Based on recent research and meta-analyses, findings on the impact of specific food groups, including fish, dairy, legumes, vegetables, and tea on the incidence of PCa have been mixed, with many studies reporting no significant association or only weak influences on overall risk (40). The relationship between fish consumption and PCa risk is complex and inconclusive, with some studies suggesting a protective effect against mortality but others linking high intake of specific fatty acids to increased incidence or lack of an association with PCa incidence. Our results support previous studies conducted in Africa, which have not found a significant relationship between dairy products and the increased risk of PCa (41, 42)Though fish is a common food in Africa, different cultures, lifestyles, and dietary patterns could influence the observed discrepancy. In addition to this, the study design employed may contribute to observed inconsistencies (26, 31). Given the conflicting opinions regarding the health benefits of fish and omega-3 fatty acids consumption and their influence on PCa, future research should take into account genetics, environmental exposure, and lifestyle choices that may have an impact on food and the development of PCa, and attempt to explore the underlying mechanisms of these relationships.

The generalizability of these associations between dietary factors and PCa risk is limited to urban Tanzanian men, as the influence of different lifestyles and environmental factors differing between urban and rural settings may influence both diet and PCa risk. Moreover, these findings necessitate cautious interpretation due to the recall bias associated with questionnaires, potential biases, and the known constraints of observational design. Limit causal conclusions. Therefore, while this study offers valuable understandings within a specific context, it highlights a need for future prospective studies employing more precise dietary measurement tools to confirm these relationships and elucidate their underlying mechanisms across diverse populations.

### 4.2 Lifestyle factors

In our study, we detected several lifestyle factors associated with positive PCa detection. In the multivariate model selection, physical activity, alcohol consumption, and tobacco smoking were important lifestyle factors associated with PCa incidence among males in the Tanzanian population. Physical activity was negatively associated with PCa incidence, while alcohol use and tobacco smoking were positively associated with the incidence of PCa among urban Tanzanian males. Our findings agree with previous research, which reported that engaging in rigorous exercise or physical activity helps maintain a healthy body weight and lowers levels of circulating insulin and inflammation markers (43, 44). Additionally, exercise strengthens the immune system and lowers oxidative stress, all of which have a preventive impact on PCa (43, 45). However, the preventive effects of physical stress are less consistent in other studies (46). This could be because of genetic changes altering the physiologic response to physical activity or variations in lifestyle factors. Our results that alcohol consumption and cigarette smoking were higher among PCa cases support previous research showing that severe consumption of alcohol and smoking cigarettes increased the risk of PCa in various populations (47, 48).

Additionally, studies suggest that men who combine both alcohol consumption and smoking cigarettes experience an increased cumulative risk due to the combined effects of DNA damage, hormonal imbalances, oxidative stress, and chronic inflammation connected to an increased risk of PCa (49). In contrast to our findings, research conducted in African communities and other demographics reported elsewhere has yielded inconsistent conclusions regarding the influence of alcohol and tobacco use on the incidence of PCa (50–53). However, participant selection, study design, and varied genetic and environmental combinations may have contributed to the inconsistent associations between alcohol consumption and PCa observed in different populations (51, 52). Thus, our study underscores the significance of lifestyle habits in reducing the incidence of PCa by promoting physical exercise and reducing cigarette smoking and alcohol consumption among males.

Truck driving, bicycle riding, were statistically significant predictors of the incidence of PCa, and in contrast, the frequency of sexual activity and motorbike riding had no influence on the incidence of cancer among patients from a bivariate model context.

### 4.3 Social demographic factors

Epidemiological evidence on the association between marital status and PCa risk remains inconsistent. In this case-control study, we found a reduced risk of PCa when widowed, divorced, and single men were combined into an unmarried category compared to married men (p < 0.05). However, when analysed separately, none of these subgroups showed a statistically significant association with PCa risk.

The revealed protective effect in the aggregated unmarried group may reflect differences in healthcare utilisation, hormonal factors, or psychosocial stress (54, 55). However, the lack of significance in individual categories suggests that this effect is not attributable to any single marital subgroup but rather emerges from their combined analysis (56). Prior studies have reported conflicting findings, with some detecting no association and others suggesting higher PCa risk among married men (55, 57–59). Conversely, Tyson et al. (60) found an elevated risk of PCa in unmarried men, though results varied across studies (60). The discrepancy between combined and stratified analyses in this study underscores the methodological complexity of marital status as a risk factor, where aggregation may conceal subgroup-specific trends (56, 61). These findings highlight the importance of analytical approach selection in epidemiological research. While combining marital status categories can enhance statistical power, it risks masking heterogeneity, such as the distinct risk profiles of widowed versus never-married men (56). Further research should explore the biological and socio-behavioural mechanisms underlying these associations.

Additionally, our findings indicate that individuals with higher education levels have an elevated risk of PCa compared to those with lower education levels. This finding appears contradictory to general health trends and some previous research. However, the discrepancy can be attributed to the surveillance effect (62), wherein individuals with higher education levels tend to be more health-conscious, undergo regular prostate-specific antigen (PSA) testing, and are more likely to receive an early diagnosis (63–65). Consequently, the observed higher incidence of PCa among well-educated individuals in our study may reflect improved diagnostic access and awareness rather than an actual increase in biological risk (63). These results align with previous studies reporting a higher incidence of PCa among individuals with advanced education levels (66).

Furthermore, our study revealed the influence of lower monthly income on the increased risk of PCa among patients seen at ORCI and MNH. The influence of lower monthly income on increased PCa risk has been previously connected to delayed diagnosis and limited access to healthcare (63). Thus, our results highlight the need for focused interventions to improve access to PCa screening and preventive healthcare for a variety of demographic groups, especially those with lower socioeconomic levels, as previously suggested (63). This result emphasises the influence of socioeconomic inequality on the course of PCa. Our research shows that Tanzanian men with lower incomes may have more difficulty getting medical care and an increased likelihood of developing PCa (67, 68). Although our findings agree with previous research, they do not corroborate studies that reported a positive association between high monthly income and increased risk of PCa.

### 4.4 Family history of cancers

In this study, the odds of being positive for PCa increased in patients with family history were a family history of PCa and those with a family history of either breast, ovarian, colorectal, endometrial, or pancreatic cancer. In line with this, several studies have reported similar results from different populations (69–72). For instance, a meta-analysis conducted in 2011 by Kiciński and colleagues revealed that males are more than twice as likely to develop PCa if they have a first-degree relative who has PCa (72). Similar findings of increased odds were reported in several previous studies, which demonstrated that men who had first-degree relatives and multiple relatives with PCa history were more likely to develop PCa compared to those with no family history of disease (69–72). We also found increased odds of PCa among patients with first-degree relatives with breast, ovarian, colorectal, endometrial, and pancreatic cancer. In agreement, several studies have demonstrated that a family history of certain cancers, particularly breast (73), ovarian (74), colorectal (75), and pancreatic (76) Cancer is associated with a higher risk of PCa.

### 4.5 Exposure to infectious and other lifestyle diseases

The current data have demonstrated the influence of gonorrhoea infection on PCa incidence; these results corroborate other findings reported elsewhere (53, 77, 78). This is because Gonorrhoea infection promotes androgen levels, chronic inflammation, and cellular damage in the prostate. Additionally, gonorrhoea infection is linked to the accumulation of free radicals and increasing oxidative stress and, therefore, DNA damage mutations, which play a significant role in PCa carcinogenesis (53, 77–79). However, other studies have not revealed the reported association due to several reasons. Variances in study design, including small sample sizes, variable population characteristics, and failure to control for confounders such as sexual behaviours and other infections, can influence results (26, 31). However, some limitations must be noted. First, this study may be subject to recall bias, as participants might not accurately report past exposures or behaviours, which could affect the reliability of the findings. Second, the unmatched case-control design may introduce selection bias, as cases and controls might not fully represent the general population, potentially limiting the generalizability of the results.

## 5 Conclusion

This study highlights the significant associations between dietary factors and the occurrence of PCa, emphasizing their potential association and predictive abilities. A comprehensive approach to risk assessment is additionally recommended to clarify these relationships. Future research, particularly prospective cohort studies and randomized controlled trials, is required to confirm and refine these observed associations. In general, our study offers significant perspectives for forthcoming research and public health initiatives aimed at reducing the incidence of PCa. These findings suggest that public health strategies incorporating dietary considerations could play a role in lowering the incidence of PCa.

## Data Availability

The original contributions presented in the study are included in the article/**Supplementary Material**. Further inquiries can be directed to the corresponding authors.

## Supplementary material

Supplementary Table 1 Bivariate models for socioeconomic and demographic factors influencing the incidence of PCa.

Supplementary Table 2 Bivariate model for lifestyle factors influencing the incidence of PCa.

Supplementary Table 3 Bivariate models for dietary patterns predicting the incidence of PCa.

Supplementary Table 4 Bivariate models for disease factors predicting the incidence of PCa.

Supplementary Table 5 Bivariate models for family history with cancer types predicting the incidence of PCa.
