# Supplementary material for: Unravelling prostate cancer risk and protective factors among urology patients in a Tanzanian population
Source: Front Oncol. 2026 Mar 19;16:1696848. doi: 10.3389/fonc.2026.1696848 (PMC13043428; doi:10.3389/fonc.2026.1696848)
Supplement: Supplementary Table 1 — Bivariate models for socioeconomic and demographic factors influencing the incidence of PCa. [file DataSheet1.docx]

**SUPPLEMENTARY MATERIALS**

**Supplementary Table S1:** Bivariate models for socioeconomic and demographic factors influencing the incidence of PCa

| Models (variables) | Estimate | Std. Error | z value | Odds Ratio | Pr(>\|z\|) | AIC |
| --- | --- | --- | --- | --- | --- | --- |
| Intercept | 0.419 | 0.182 | 2.300 | 1.52 | 0.0215 |  |
| Other cf. Married | -1.487 | 0.394 | -3.770 | 0.23 | 0.0002 | 222.2 |
| Intercept | 0.436 | 0.199 | 2.190 | 1.55 | 0.0280 |  |
| Monthly income | -0.580 | 0.196 | -2.960 | 0.56 | 0.0030 | 228.1 |
| Intercept | -3.305 | 1.388 | -2.380 | 0.04 | 0.0170 |  |
| Age (years) | 0.048 | 0.020 | 2.440 | 1.05 | 0.0150 | 231.1 |
| Intercept | -0.704 | 0.338 | -2.090 | 0.49 | 0.0370 |  |
| Number of children | 0.160 | 0.063 | 2.530 | 1.17 | 0.0120 | 231.2 |
| Intercept | -0.560 | 0.627 | -0.890 | 0.57 | 0.3700 |  |
| Children present cf. Children absent | 0.661 | 0.647 | 1.020 | 1.94 | 0.3100 | 237 |
| Intercept | 0.583 | 0.658 | 0.890 | 1.79 | 0.3800 |  |
| Body Mass Index (BMI) | -0.022 | 0.027 | -0.820 | 0.98 | 0.4100 | 237.4 |
| Intercept | 0.091 | 0.302 | 0.300 | 1.10 | 0.7600 |  |
| Lower education cf. Higher Education | -0.043 | 0.351 | -0.120 | 0.96 | 0.9000 | 238.1 |
| Intercept | 0.074 | 0.385 | 0.190 | 1.08 | 0.8500 |  |
| Business entrepreneur cf. Agriculture | 0.149 | 0.511 | 0.290 | 1.16 | 0.7700 | 241.9 |
| Employed cf. Agriculture | -0.560 | 0.592 | -0.950 | 0.57 | 0.3400 |  |
| Peasant cf. Agriculture | -0.143 | 0.535 | -0.270 | 0.87 | 0.7900 |  |
| Retiree cf. Agriculture | 0.141 | 0.470 | 0.300 | 1.15 | 0.7600 |  |

**Supplementary Table S2:** Bivariate model for lifestyle factors influencing the incidence of PCa

|  | Estimate | | Std. Error | | z value | Pr(>\|z\|) | Odds Ratio | AIC |
| --- | --- | --- | --- | --- | --- | --- | --- | --- |
| Intercept | | -0.702 | | 0.202 | -3.48 | 0.001 | 0.50 | 195.5 |
| Alcohol | | 1.012 | | 0.192 | 5.27 | 0.000 | 2.75 |  |
| Intercept | | 0.960 | | 0.267 | 3.59 | 0.000 | 2.61 |  |
| Physical activities | | -0.662 | | 0.154 | -4.29 | 0.000 | 0.52 | 218.6 |
| Intercept | | -0.334 | | 0.183 | -1.82 | 0.068 | 0.72 |  |
| Tobacco smoking | | 0.697 | | 0.191 | 3.65 | 0.000 | 2.01 | 223.4 |
| Intercept | | 0.156 | | 0.162 | 0.97 | 0.334 | 1.17 |  |
| Track driving | | -1.255 | | 0.600 | -2.09 | 0.036 | 0.29 | 234.5 |
| Intercept | | 0.336 | | 0.210 | 1.60 | 0.110 | 1.40 |  |
| Bicycle riding | | -0.262 | | 0.130 | -2.02 | 0.043 | 0.77 | 235.4 |
| Intercept | | 0.407 | | 0.258 | 1.58 | 0.115 | 1.50 |  |
| Sexual activity | | -0.253 | | 0.145 | -1.75 | 0.081 | 0.78 | 236.5 |
| Intercept | | 0.074 | | 0.1631 | 0.46 | 0.650 | 1.08 |  |
| Motor bike riding | | -0.076 | | 0.1547 | -0.49 | 0.620 | 0.93 | 239.3 |

**Supplementary Table S3 :** Bivariate models for dietary patterns predicting the incidence of PCa

| Variable | Estimate | Std. Error | z value | Pr(>\|z\|) | Odds Ratio | AIC |
| --- | --- | --- | --- | --- | --- | --- |
| Intercept | -2.219 | 0.379 | -5.86 | 0.0000 | 0.11 |  |
| Red meat | 1.510 | 0.230 | 6.56 | 0.0000 | 4.53 | 165.4 |
| Intercept | 1.436 | 0.264 | 5.43 | 0.0000 | 4.20 |  |
| Tomatoes | -1.543 | 0.224 | -6.87 | 0.0000 | 0.21 | 168.1 |
| Intercept | 1.237 | 0.284 | 4.35 | 0.0000 | 3.44 |  |
| Coffee | -0.743 | 0.142 | -5.23 | 0.0000 | 0.48 | 206.9 |
| Intercept | -0.760 | 0.242 | -3.14 | 0.0017 | 0.47 |  |
| Food containing fats | 0.793 | 0.183 | 4.33 | 0.0000 | 2.21 | 217.8 |
| Intercept | 1.905 | 0.495 | 3.85 | 0.0001 | 6.72 |  |
| Soya | -0.813 | 0.202 | -4.02 | 0.0001 | 0.44 | 220.4 |
| Intercept | -2.356 | 0.856 | -2.75 | 0.0059 | 0.09 |  |
| grains | 0.701 | 0.244 | 2.87 | 0.0041 | 2.01 | 230.6 |
| Intercept | -0.016 | 0.157 | -0.1 | 0.9200 | 0.98 |  |
| Vitamin-mineral supplements | 0.768 | 0.517 | 1.48 | 0.1400 | 2.16 | 235.9 |
| Intercept | 0.517 | 0.361 | 1.43 | 0.1500 | 1.68 |  |
| Fish | -0.213 | 0.148 | -1.44 | 0.1500 | 0.81 | 237.5 |
| Intercept | -0.335 | 0.311 | -1.08 | 0.2800 | 0.72 |  |
| Dairy food | 0.215 | 0.153 | 1.41 | 0.1600 | 1.24 | 237.6 |
| Intercept | 0.808 | 0.481 | 1.68 | 0.0930 | 2.24 |  |
| Vegetables | -0.255 | 0.152 | -1.68 | 0.0930 | 0.77 | 236.7 |
| Intercept | 0.319 | 0.537 | 0.59 | 0.5500 | 1.38 |  |
| Legumes | -0.105 | 0.199 | -0.53 | 0.6000 | 0.90 | 239.3 |
| Intercept | 0.116 | 0.521 | 0.22 | 0.8200 | 1.12 |  |
| Green and black tea | -0.024 | 0.171 | -0.14 | 0.8900 | 0.98 | 239.6 |

**Supplementary Table S4:** Bivariate models for disease factors predicting the incidence of PCa

|  | **Estimate** | | **Std. Error** | | **z value** | **Pr(>\|z\|)** | **Odds Ratio** | **AIC** |
| --- | --- | --- | --- | --- | --- | --- | --- | --- |
| (Intercept) | | -0.090 | | 0.161 | -0.56 | 0.574 | 0.914 |  |
| Gonorhea | | 1.962 | | 0.776 | 2.53 | 0.011 | 7.115 | 230.3 |
|  | |  | |  |  |  |  |  |
| (Intercept) | | 0.207 | | 0.172 | 1.20 | 0.231 | 0.988 | 235.2 |
| Diabetic | | -0.813 | | 0.398 | -2.04 | 0.041 | 3.544 |  |
|  | |  | |  |  |  |  |  |
| (Intercept) | | -0.012 | | 0.158 | -0.08 | 0.940 | 0.988 |  |
| Syphyllis | | 1.265 | | 0.817 | 1.55 | 0.120 | 3.544 | 236.7 |
|  | |  | |  |  |  |  |  |
| (Intercept) | | -0.042 | | 0.167 | -0.25 | 0.800 | 0.959 |  |
| Erectile Dysfunction | | 0.573 | | 0.432 | 1.32 | 0.190 | 1.773 | 237.8 |

**Supplementary Table S5**: Bivariate models for family history with cancer types predicting the incidence of PCa

|  | **Estimate** | **Std. Error** | **z value** | **Pr(>\|z\|)** | **Odds_Ratio** | **AIC** |
| --- | --- | --- | --- | --- | --- | --- |
| (Intercept) | -0.214 | 0.169 | -1.26 | 0.20737 | 0.807692 | 220.2 |
| Family history with prostate cancer | 1.918 | 0.569 | 3.37 | 0.00075 | 6.809524 |  |
|  |  |  |  |  |  |  |
| (Intercept) | -0.0662 | 0.1628 | -0.41 | 0.684 | 0.935897 | 231.2 |
| Family history of any type of cancer | 1.1649 | 0.5999 | 1.94 | 0.052 | 3.205479 |  |
